# The Impact of Gut Microbiome on Metabolic Disorders During Catch-Up Growth in Small-for-Gestational-Age

**DOI:** 10.3389/fendo.2021.630526

**Published:** 2021-03-04

**Authors:** Jingjing An, Junqi Wang, Li Guo, Yuan Xiao, Wenli Lu, Lin Li, Lifen Chen, Xinqiong Wang, Zhiya Dong

**Affiliations:** ^1^ Department of Endocrine and Metabolic Disease, Ruijin Hospital, School of Medicine, Shanghai Jiao Tong University, Shanghai, China; ^2^ Department of Pediatrics, Ruijin Hospital, School of Medicine, Shanghai Jiao Tong University, Shanghai, China; ^3^ Molecular Medicine Program, University of Utah, Salt Lake City, UT, United States

**Keywords:** catch-up growth, gut microbiome, *Lactobacillus*, short-chain fatty acids, small for gestational age

## Abstract

**Objective:**

Catch-up growth (CUG) in small for gestational age (SGA) leads to increased risk of metabolic syndrome and cardiovascular diseases in adults. It remains unclear if microbiota could play an important role in CUG-SGA independent of genetic or nutritional factors. The present study explored the role of gut microbiota in, and its association with, metabolic disorders during CUG-SGA.

**Methods:**

An SGA rat model was established by restricting food intake during pregnancy, and the rats were divided into catch-up growth (CUG-SGA) and non-catch-up growth (NCUG-SGA) groups based on body weight and length at the fourth postnatal week. High-throughput sequencing of 16S rRNA was conducted to detect the diversity and composition of the gut microbiota. Fecal short-chain fatty acids (SCFAs) were detected by gas chromatography-mass spectrometry. Transcriptome sequencing of liver tissue was performed and verified using real-time PCR. Concentrations of insulin and total cholesterol were determined using enzyme-linked immunosorbent assay.

**Results:**

The composition of gut microbiota in CUG-SGA rats differed from that of NCUG-SGA rats, with reduced abundance of *Lactobacillus* in the CUG-SGA group. The decrease in *Lactobacillus* was significantly associated with increased body weight and upregulated insulin and total cholesterol levels. Five SCFAs and two branched chain fatty acids were significantly higher in the CUG-SGA group than in the NCUG-SGA group. Additionally, SCFAs were positively associated with clinical indices such as weight, body mass index, insulin, and total cholesterol. Transcriptomic data revealed that insulin-like growth factor-2 expression was significantly decreased in CUG-SGA rats and was associated with a decrease in *Lactobacillus* bacteria.

**Conclusion:**

*Lactobacillus* and SCFAs were associated with the metabolic disorders during CUG in SGA. Gut microbiome may play a certain role on metabolic disorders during catch-up growth in small-for-gestational-age.

## Introduction

Small for gestational age (SGA) refers to newborns whose birth weight and/or length are at least 2 standard deviations (SDs) below the mean for the gestational age (≤−2 SD) (1). SGA is a common medical problem with a worldwide incidence of 2.3%–10% (2). Newborns with SGA often require additional medical care, including temperature-controlled incubators, tube feeding, and monitoring of blood glucose levels. With advances in medical care, the mortality rate of newborns with SGA has significantly decreased. However, survivors are at increased risks of developing metabolic syndrome and cardiovascular diseases in adulthood compared with infants with normal birth weight (3, 4).

About 85%–90% of infants with SGA demonstrate catch-up growth (CUG) during the first two years of life (5). Worldwide epidemiological data have revealed that CUG of SGA infants may contribute to insulin resistance (6). Eriksson et al found that some SGA infants caught up rapidly after birth, reaching or exceeded the normal body mass index level of children of the same age by 7 years, but with a high death rate associated with cardiovascular disease (7). A cohort study in India found similar results. They found that insulin resistance indicators, total lipoproteins, and low-density lipoproteins were higher in such group of infants (8). However, the relevant mechanism remains unclear.

In the last two decades, our understanding of the causes of SGA has significantly improved, although it still remains limited. For example, fetal genetic deficiencies in the growth hormone/insulin-like growth factor-1 axis have been discovered in SGA (9, 10). Several single nucleotide polymorphisms have been associated with diabetes or obesity in patients born with SGA (9, 11) and maternal placental gene mutations can also cause SGA (12). In addition to genetic factors, other factors such as environmental factors can cause SGA, although the mechanism remains elusive. Whether the intestinal microbiota can impact the host’s metabolic activity and contribute to SGA also remains unclear.

In recent years, gut microbiota has been found to be an important environmental factor in the regulation of host metabolism and to significantly impact body weight. Studies have found that the fat content of conventionally raised mice is 40% higher than that of Germ free (GF) mice, independent of food intake (13). When the intestinal microbiota of normal mice is transplanted into GF mice, it changes the body fat composition of the latter (13, 14), suggesting that the intestinal microbiota is involved in regulating the host's ability to obtain energy from the diet. Another study found that transplanting the intestinal microbiota of lean donors into patients with metabolic syndrome improved their insulin sensitivity, implying that the intestinal microbiota is involved in the control of body weight and insulin resistance (15). The intestinal microbiota is involved in the metabolism and transformation of a variety of nutrients, including carbohydrates, proteins, and fats. Studies have found that the intestinal microbiota transforms the indigestible carbohydrates into monosaccharides and short-chain fatty acids (SCFAs). Meanwhile, SCFAs can be used for de novo synthesis of lipids and glucose, thereby affecting the host's glucose and lipid metabolism (16). A cross-sectional study found that the fecal SCFA concentration of overweight or obese subjects is significantly higher than that of thinner controls, and higher SCFA levels have a good correlation with metabolic syndrome indicators (17–20). Studies on genetically obese mice have shown that the intestinal microbiota increases energy utilization by producing excessive SCFAs, thereby affecting the host's metabolism (14). Therefore, we hypothesized that the intestinal microbiota and its metabolites play an important role in host metabolism during the CUG stage in SGA.

We established an SGA rat model and divided these rats into two groups based on their body weight and length —catch-up growth (CUG -SGA) and without catch-up growth (non-catch-up growth, NCUG -SGA). Further, we compared the composition of gut microbiota in these groups. Since the liver is the major metabolic organ, to further verify our hypothesis, we sequenced the transcriptome of liver tissues to identify metabolism-related genes in CUG- and NCUG-SGA rats. The findings of our study will further our understanding of SGA in humans.

## Materials and Methods

### Animal Model

A rat model of SGA was established in animals under maternal undernutrition during pregnancy as previously described (21, 22). In brief, adult SPF-grade Sprague-Dawley female (n=15) and male (n=5) rats were mated overnight. The pregnant rats were randomly divided into SGA group (n=10) and normal control group (n=5). Pregnant rats in the normal control group had standard food diet available *ad libitum* throughout pregnancy, while pregnant rats in the SGA group were under food restriction, with 50% reduction of food intake (about 10 g) from the first day after conception. Rats in both groups gave birth naturally. All newborns were breastfed for the first 3 weeks after birth, and then fed with standard chow. The litter size was culled to five pups per litter at birth in the SGA group and the normal control group, to ensure the catch-up growth of the offspring with SGA. Newborns were defined as SGA rats when the birth weight and length were -2SD below those of the newborns in the normal control. Body weight and length were monitored weekly. NCUG-SGA rats were identified when the body weight and body length were -2SD lower than those in the normal group at week 4 following parturition, and the rest were labelled as CUG-SGA rats. All procedures in this study were approved by the animal experiment ethics committee of Ruijin Hospital, Shanghai Jiao Tong University School of Medicine.

### Enzyme-Linked Immunosorbent Assay (ELISA)

Commercial ELISA kits were used to measure fasting insulin (Crystal Chem, Illinois, USA), IGF-1 (Crystal Chem, Illinois, USA), and total cholesterol (Zhuocai Biological, Shanghai, China), following the manufacturers’ protocols.

### Fecal Genomic DNA Extraction and 16S rRNA High-Throughput Sequencing 

Five 4-week-old pups were randomly selected from each of the two groups (10 in total, all from separate cages), and their feces collected for 16S rRNA sequencing and SCFA quantification.

Fecal genomic DNA was extracted using TIA Namp Stool DNA Kit (Invitrogen, California, USA) according to the manufacturer’s protocol. DNA concentration and integrity were determined using gel electrophoresis. PCR amplification of the bacterial 16S rRNA genes V3–V4 region was performed using the forward primer 520F (5'- AYTGGGYDTAAAGNG -3') and the reverse primer 802R (5'- TACNVGGGTATCTAATCC -3'). Thermal cycling consisted of initial denaturation at 98°C for 5 min, followed by 25 cycles consisting of denaturation at 98°C for 30 s, annealing at 53°C for 30 s, and extension at 72°C for 45 s, with a final extension of 5 min at 72°C. PCR products were purified with AmpureXp beads (AGENCOURT), diluted, denatured into single-strands in NaOH buffer, and sequenced on an Illumina Hiseq2500 platform. The raw sequence reads were then processed based on sequence quality. After quality control, the raw reads were classified based on index and barcode, and clustered into operational taxonomic units using QIIME2 dada2 or Vsearch software (https://docs.qiime2.org/2019.7/citation/). Sequencing data analyses were mainly performed using QIIME2 and R packages (v3.2.0). ASV-level alpha diversity indices, such as Chao1 richness estimator, were calculated using the ASV table in QIIME2, by Kruskal-Wallis test, and visualized as box plots. Wilcoxon rank-sum test was utilized to detect differentially enriched microbiota at genus between NCUG-SGA and CUG-SGA groups (P < 0.05). Beta diversity analysis was performed to investigate the structural variation of microbial communities among different samples using Bray-Curtis metrics and visualized *via* principal coordinate analysis (PCoA). Non-parametric permutational multivariate analysis of variance (PERMANOVA) was conducted for analyzing significant difference of microbiota structure between the two groups. LEfSe (Linear discriminant analysis effect size) was performed to detect differentially abundant taxa in both groups (23). Microbial functions were predicted by PICRUSt2 (Phylogenetic investigation of communities by reconstruction of unobserved states) upon KEGG (https://www.kegg.jp/) databases (24).

### Fecal SCFA Detection

Feces from the 4-week-old rats were thawed on ice, and approximately 10 mg of feces were homogenized in 50 μl of 15% phosphoric acid. The suspensions were homogenized with a vortex and centrifuged at 12,000 rmp for 10min at 4°C. The supernatants were processed for gas chromatography-mass spectrometry on an Agilent 7890A/5975C mass spectrometer (Agilent Technologies, California, USA). The injection volume was 1 μl, and the split ratio was 10:1. Samples were separated with an Agilent HP-INNOWAX capillary GC column (30 m × 0.25 mm ID × 0.25 µm). The initial temperature was 90°C and was increased to 120°C at 10°C/min, after which the temperature was increased to 150°C at 5°C /min and then to 250°C at 25°C /min, where it remained for 2 min. The carrier gas was helium (1.0 ml/min). The temperatures of the injection port and ion source were 250°C and 230°C respectively under SIM model. Agilent MSD ChemStation software was used for quantitative analysis of chromatographic peak area and retention time (25).

### RNA Extraction, Library Construction, Transcriptome Sequencing and Bioinformatics Analysis

Rat liver tissues were stored in liquid nitrogen prior to homogenization with a pestle and total RNA was extracted using Trizol reagent (Invitrogen, California, USA) according to the manufacturer’s instructions. RNA integrity was analyzed *via* gel electrophoresis using 1% agarose gel. RNA concentration and quality were measured using a Nanodrop 2000 spectrophotometer (Thermo, Massachusetts, USA). RNA samples (3 μg) were used for library construction using a TruSeq RNA kit (Illumina, California, USA), following the manufacturer’s protocol. Clone library was quality-checked using an Agilent 2100 Bioanalyzer (Agilent Technologies, California, USA) and processed for Paired-end sequencing using Next-Generation Sequencing technology on the Illumina MiSeq platform (Illumina, California, USA).

After sequencing, the images were first converted into raw data in FASTQ format and filtered using Cutadapt (v1.15) software to obtain high-quality reads (clean reads) and analyzed for Q20, Q30 and GC. The filtered clean reads were then mapped to the reference genome using the HISAT2 software (http://ccb.jhu.edu/software/hisat2/index.shtml) to obtain CDS (coding region), Intron (intron), Intergenic (intergenic region) and UTR (5' and 3' untranslated regions) sequences. Gene expression was calculated using the HTSeq Read Count and those with a normalized FPKM value >1 were considered to be expressed. DESeq analysis of RNA-Seq data was set as log2 fold change > 1 or <-1 and P value < 0.05. The volcano map of DEG was generated using the “ggplots2” package in R. DAVID software (6.8) was used for GO and KEGG pathway analysis of the differentially expressed genes. A protein-protein interaction (PPI) network was constructed *via* STRING (Search3 Tool for the Retrieval of Interacting Genes/Proteins), and visualized using Cytoscape software.

### RNA-Seq Validation by qRT-PCR

To verify the accuracy of RNA-Seq, Igf2, Mmp14, and Hgf were selected from among the differentially expressed genes, with glyceraldehyde 3-phosphate dehydrogenase (GAPDH) as an internal reference. After being transcribed into cDNA, PCR was performed on a TIB8600 system (Taipu Biosciences, China) using a 2×SYBR real-time PCR premixture reagent. The primers were designed using Primer Premier 5.0 software and synthesized by Personalbio, Shanghai, China. The relative expression of each gene was calculated using the 2^-△△Ct^ method and data were expressed as the mean ± SD. The specific primers used are shown in S1.

### Statistical Analysis

GraphPad Prism 8.0 (GraphPad Software, California, USA) and SPSS 23.0 (IBM, Chicago, USA) were used for analyzing data and drawing charts. Normally distributed data was expressed as the mean ± standard deviation (X ± SD). Pairwise differences between groups were analyzed using *t* test. Correlation analysis was performed using Spearman correlation analysis. Correlation coefficient (r) values > 0.6 were considered to represent strong correlations. P values lower than 0.05 were considered significant: *p ≤ 0.05, **p ≤ 0.01, ***p ≤ 0.001.

## Results

### Characteristics of Rats With NCUG-SGA and CUG-SGA

**Figure 1:**
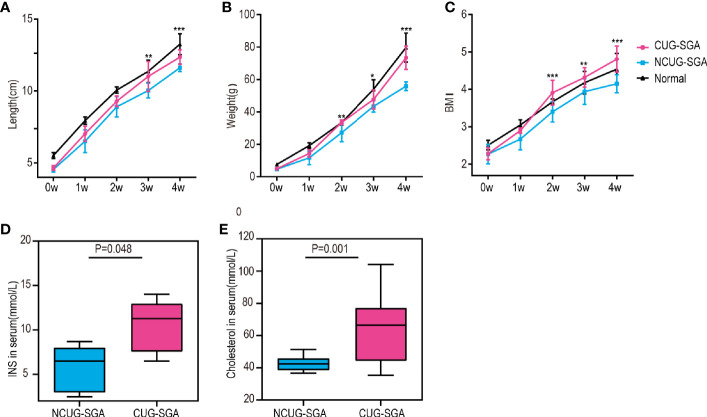
General characteristics of rats between catch-up growth in small for gestational age (CUG-SGA) and non-catch-up growth in small for gestational age (NCUG-SGA) groups **(A)** Body length, **(B)** body weight, and **(C)** Body mass index (BMI) were tracked weekly. Insulin (INS) **(D)** and total cholesterol **(E)** were measured at week 4. CUG-SGA (n=13) and NCUG-SGA(n=12). *p< 0.5, **p < 0.01, ***p < 0.001 (NCUG-SGA vs CUG-SGA).

We first characterized the SGA model in our study. Normal pups are born with normal body weight and length . As described in the methods above, SGA rats are defined as body weight and body length -2SD below the rats from control group at birth, i.e. rats appropriate for gestational age at birth. There were 70 offspring of pregnant rats in the SGA group with restricted diet, including 8 normal weight pups and 62 SGA pups. To ensure catch-up growth of the SGA pups, the litter size was culled to five pups per litter at birth, a total of 50 SGA rats were left. All newborns were breastfed for the first 3 weeks after birth, and then fed standard food. Although given the same food, 26 SGA rats exhibited CUG while other 24 did not. NCUG-SGA rats were identified by week 4, when their body weights were -2SD lower than those in the normal group, and the rest of the SGA rats were defined as CUG-SGA. There were significant differences in the body weight, length , and BMI between rats with CUG-SGA and NCUG-SGA (**Figures 1A–C**). Thus, 4-week-old rats were used in further investigations.

Since CUG in SGA has been associated with increased insulin resistance and metabolic syndrome in humans, we measured if this is consistent with our rat model. Serological analysis showed that both fasting insulin (INS) and total cholesterol concentrations in CUG-SGA rats were higher than those in the NCUG-SGA rats (**Figures 1D, E**).

### Differences in the Gut Microbiota Between Rats With NCUG-SGA and CUG-SGA as Revealed by 16S rRNA Sequencing

**Figure 2:**
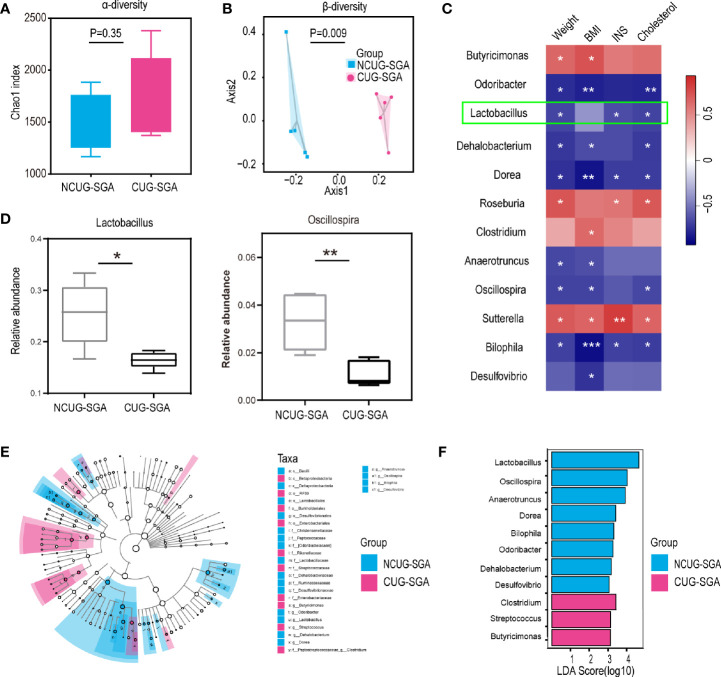
Variations in intestinal microbiota between non-catch-up growth in small for gestational age (NCUG-SGA) and catch-up growth in small for gestational age (CUG-SGA) rats **(A)** The α diversity of the intestinal microbiota of rats between the two groups (Chao1 index) showed that both groups have variations among individual samples within each group, but not significant difference between the two groups; **(B)** Comparison of the β diversity of the intestinal microbiota based on the Bray-Curtis distance shows significant difference between the NCUG- and CUG-SGA rats; **(C)** A heatmap of Spearman correlation between microbiota at genus level and clinical characteristics; **(D)** Box plots showing the abundance of Lactobacillus and Bilophila in the NCUG-SGA and CUG-SGA groups; **(E)** Differences of classification unit between the groups; **(F)** Linear discriminant analysis (LDA) effect size algorithm (LEfSe) of intestinal microbiota (LDA>3).

To compare the microbiota profiles between the NCUG-SGA and CUG-SGA rats, we performed 16S rRNA high-throughput sequencing on rat feces. We first compared the microbiota diversity of individual samples, as indicated by the α diversity index (**Figure 2A**). In both groups, we observed relatively diverse microbiota and variations within each group, without significant difference between NCUG-SGA and CUG-SGA. When we compared the microbiota between the two groups, as indicated by the β diversity index, gut microbiota clearly clustered separately in the NCUG-SGA and CUG-SGA groups, indicating that despite individual variations within the same group, the composition of the gut microbiota differed in these two groups (**Figure 2B**). We further ran the linear discriminant analysis (LDA) effect size algorithm (LEfSe) analysis to evaluate the classification difference of the microbiota between the NCUG-SGA and CUG-SGA groups (**Figures 2D–F**).

It is possible that the difference in microbiota could be the driving force for the differences observed in host growth and metabolism. Therefore, we used the relative abundance results to identify the genera of gut microbiota that are associated with body weight, BMI, INS, and total cholesterol. As shown in **Figure 2C**, *Butyricimonas, Roseburia, Clostridium*, and *Sutterella* were positively correlated with body weight, BMI, INS level, and/or total cholesterol level respectively, while *Odoribacter*, *Lactobacillus*, *Dehalobacterium*, *Dorea, Anaerotruncus, Oscillospira, Bilophila*, and *Desulfovibrio* were negatively associated with body weight, BMI, INS and/or cholesterol level, suggesting that among the thousands of bacteria detected, these 12 different genera may play an important role in shaping the host body weight and metabolic activity.

### Fecal SCFA Content Is Higher in the CUG-SGA Group Than in the NCUG-SGA Group

**Figure 3:**
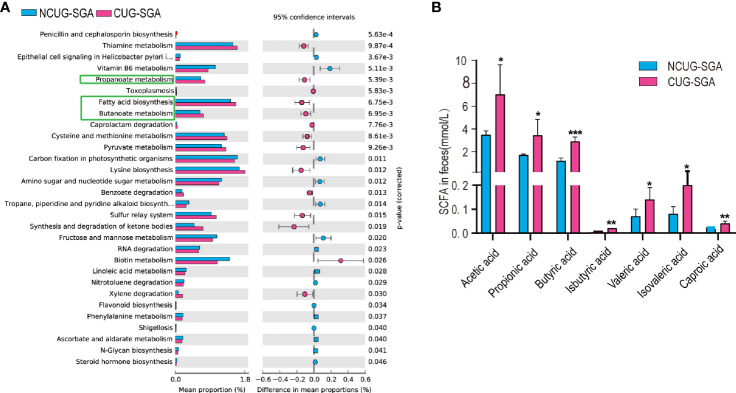
Intestinal microbiota functional prediction and short-chain fatty acid (SCFA) detection. **(A)** KEGG pathway analysis based on 16S rRNA between NCUG-SGA and CUG-SGA groups in rats, among them, fatty acid biosynthesis, propionate metabolism, and butanoate metabolism are significantly upregulated in the CUG-SGA group rats. **(B)** Detection of SCFA from fecal samples in NCUG-SGA and CUG-SGA groups. *p < 0.5, **p < 0.01, ***p < 0.001. CUG-SGA, catch-up growth in small for gestational age; NCUG-SGA, non-catch-up growth in small for gestational age.

Gut microbiota and their metabolic products play a critical role in shaping host metabolism and growth, as shown by the defects observed in GF animals (13, 14). However, the16s rRNA sequencing data does not provide functional profile directly. To further understand the potential effect of the microbiota on host in SGA, we used PICRUSt2 for functional prediction. We found that the abundance of 30 KEGG pathways was significantly different between the NCUG-SGA and CUG-SGA groups. The pathways enriched in the CUG-SGA group include the pathways of fatty acid biosynthesis, propionate metabolism, and butanoate metabolism, which implied that SCFAs may play an important role in the mechanism of CUG and its metabolic change (**Figure 3A**).

SCFAs, one of the main metabolites of the gut microbiota, play a role in energy regulation and metabolism by acting as intermediaries between the gut microbiota and the host. To examine the potential role of SCFAs in regulating host metabolism during CUG, we first measured SCFA concentrations in the feces of the rats at four weeks of age. As shown in **Figure 3B**, there were five SCFAs (acetic acid, propionic acid, butyric acid, valeric acid and caproic acid) and two branched chain fatty acids (iso butyric acid, isovaleric acid), that were significantly higher in the feces of rats in the CUG-SGA group than in the NCUG-SGA group (all p values < 0.05), which was consistent with the prediction results of intestinal microbiota function.

### SCFAs Are Associated With the Gut Microbiota and Clinal Features in SGA 

**Figure 4:**
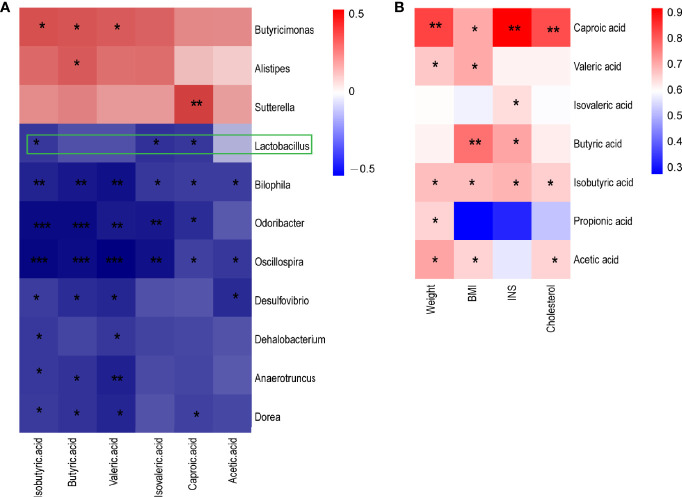
A heatmap of Spearman correlation between intestinal microbiota at genus level and metabolism index and SCFA **(A)** A heatmap of Spearman correlation between microbiota at genus level and SCFAs. **(B)** A heatmap of Spearman correlation between clinical characteristics and SCFAs. Red: positive correlation; blue: negative correlation; *P < 0.05, **P < 0.01, ***P < 0.001. SCFAs, short-chain fatty acids.

The function prediction of gut microbiota and SCFA levels suggested that the gut microbiota may play an important role in metabolic syndrome in CUG in SGA through SCFA. First, we established a correlation matrix based on the Spearman rank correlation coefficient to explore the relationship between intestinal microbiota and fecal SCFA levels. Eleven different bacterial genera were significantly related to at least one SCFA (P < 0.05, |r|>0.6), indicating that SCFA levels were closely related to the intestinal microbiota. Among them, *Lactobacillus*, *Bilophila*, *Oscillospira*, and *Desulfovibrio* had a significant negative correlation with different SCFAs (P < 0.05, r <-0.6). The concentration of butyric acid was positively correlated with the abundance of butyric acid-producing *Butyricimonas* (P < 0.05, r> 0.6) (**Figure 4A**).

Spearman correlation analysis was performed to determine the correlation between the clinical indicators of NCUG-SGA and CUG-SGA rats and SCFAs. The results showed that five SCFAs and two branched chain fatty acids had a significant positive correlation with at least one clinical index (weight, BMI, insulin, and total cholesterol), indicating that SCFAs may affect the metabolism of the host (**Figure 4B**). These findings suggest that gut microbiota and SCFAs may play a role in the metabolic syndrome associated with the catch-up growth of SGA.

### Changes in Metabolism-Related Pathways in the Liver Transcriptome in CUG-SGA Rats With Decreased Igf2 Expression

**Figure 5:**
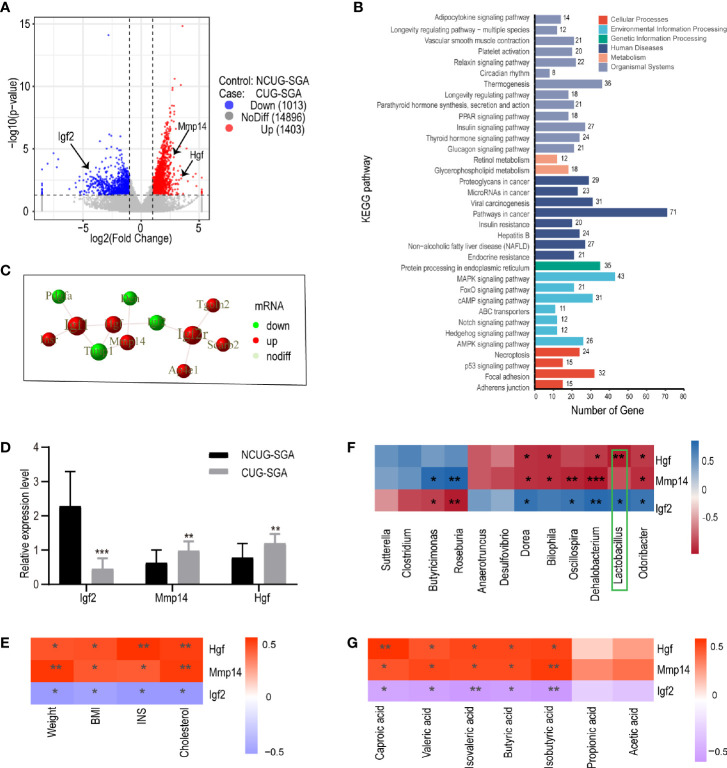
Transcriptome sequencing, validation, and correlation analysis of metabolism-related genes with microbiota and clinical indicators in liver tissues of rats from NCUG-SGA (n=5) and CUG-SGA (n=5) groups **(A)** Volcano plots of differentially expressed genes (DEGs) with |log2FoldChange|>1 and P value<0.05; **(B)** KEGG pathways of DEGs; **(C)** the protein-protein interaction (PPI) of Igf2, Mmp14, and Hgf; **(D)** qRT-PCR validation of metabolism-related genes; **(E)** Correlation analysis between metabolism-related genes and clinical indicators; **(F)** Correlation analysis between metabolism-related genes and microbiota at genus level; **(G)** Correlation analysis between metabolism-related genes and SCFA. *P < 0.05, **P <0.01, ***P< 0.001.

The study on the gut microbiota of SGA rats indicated that SCFAs may be involved in the metabolic disorders in the process of catch-up growth. The liver is an important metabolic organ in mammals, and it establishes connections with other tissues of the body through its metabolic function. To clarify the correlation between SCFAs and the metabolic disorders associated with CUG in SGA, we sequenced the transcriptome of liver tissues in NCUG-SGA and CUG-SGA rats. Results revealed 2416 differentially expressed genes (DEGs), of which 1403 were upregulated and 1013 were downregulated (**Figure 5A**). To clarify the biochemical metabolism and signal transduction pathways involved in DEGs, we conducted KEGG pathway analysis and found significant differences in 53 metabolic pathways (P<0.05). **Figure 5B** shows the top 34 metabolic pathways, including protein synthesis, fatty acid metabolism, AMPK signaling, insulin signaling, PPAR signaling, MAPK signaling, and insulin resistance. These results provide important clues for studying the metabolic syndrome in CUG in SGA.

To systematically analyze the functions of DEGs in the liver tissues of the NCUG-SGA and CUG-SGA groups, we mapped the DEGs to the protein-protein interaction (PPI) (https://string-db.org/) database and generated the PPI network diagram **(S2)**. Igf2, Mmp14, and Hgf were significantly different between the two groups, and showed interactions with each other (**Figure 5C**), Igf2 was very close to the gene encoding insulin on human chromosome 11p, which may contribute to regulate the body weight and obesity in children and adults (26); Mmp14 and Hgf were also associated with insulin resistance (27, 28), and these three genes are members of MAPK pathway mentioned above. Igf-2 was significantly downregulated in the CUG-SGA group. The expression of Hgf and Mmp14 significantly increased in the CUG-SGA group. These results were verified using qRT-PCR, indicating that transcriptome sequencing can reliably reflect the changes in gene expression (**Figure 5D**).

Correlation analysis showed that Igf2, Hgf, and Mmp14 were significantly correlated with clinical metabolic indicators (weight, BMI, INS, and TC) (P<0.05, |r|>0.6) (**Figure 5E**), indicating that these three genes may be involved in metabolic changes during SGA growth catch-up. To clarify the correlation between DEGs and intestinal microbiota , we analyzed the correlation between Igf2, Hgf, and Mmp14 and the gut microbiota and SCFA. The results showed that Igf2 and Hgf were significantly related to the abundance of *Lactobacillus* (P<0.05, |r|>0.7) (**Figure 5F**); the expression levels of Igf2, Hgf, and Mmp14 were related to the changes in SCFA (P<0.05, |r|>0.6) (**Figure 5G**).

## Discussion

Insulin resistance and metabolic syndrome greatly affect human life expectancy and quality of life. A large number of studies have found that the risk of insulin resistance and obesity is significantly increased in the growth of SGA individuals, and the metabolic disorders are related to CUG. In this study, we aimed to evaluate the role of gut microbiota independent of genetic factors in the process of CUG in SGA. Pregnant rats were given restricted food (reduced by 50% from the first day of gestation until delivery as compared to normal diet) to give birth to SGA pups. At 4 weeks, the length and weight of CUG-SGA rats were significantly higher than those in the NCUG-SGA group, with elevated levels of insulin and total cholesterol. This was associated with significant differences in the diversity and composition of gut microbiota between these two groups, including *Lactobacillus*, whose relative abundance was most significantly decreased in the CUG-SGA group. SCFA levels in the CUG-SGA group were significantly increased, with significant changes in the mRNA level of IGF-2 as shown by the liver transcriptome analysis. Our findings suggest that the gut microbiota and its metabolites may relate to the metabolic process in the liver and may be associated with increased body weight and the systemic metabolic dysfunction in the host.

To study the relationship between gut microbiota and host metabolism, we used different techniques to capture and present a complete and unbiased picture, including 16s rRNA sequencing, SCFA detection, and liver tissue RNA sequencing. This allowed us to identify the decrease in *Lactobacillus* as the most significant event across different analyses in CUG-SGA as compared to the NCUG-SGA group. The gram-positive *Lactobacillus* is the dominant bacteria in the gastrointestinal microbiota of mammals and can affect the composition and metabolism of gut microbiota by producing metabolites such as lactic acid which lowers pH (29, 30). The impact of *Lactobacillus* on weight change varied according to the species: some species were associated with weight gain, while others were linked to weight loss. Meta-analyses have shown that *Lactobacillus* can promote loss of weight and fat in overweight adults and children (31, 32). *Lactobacillus* strains isolated from the feces of super-longevity people reduced serum cholesterol levels in rats with a high-cholesterol diet (33). *Lactobacillus* selected from fermented cow milk also reduced serum cholesterol levels in rats (34). Moreover, the effect of the same strain of Lactobacillus could be different depending on the species of the host (35). In our current study, the increase in body weight, insulin, and total cholesterol in the CUG group was significantly associated with decreased abundance of *Lactobacillus*. Therefore, the metabolic disorder of catch-up of SGA may be related to the decrease in *Lactobacillus* and other bacteria.

Furthermore, our study explored the changes of metabolites such as SCFAs. PICRUSt2s functional prediction based on 16S rRNA sequencing data showed that CUG-SGA rats microbiome were associated with metabolic pathways including fatty acid, propionate, butyrate, etc. In consistency, the contents of fecal SCFAs were significantly higher in the CUG-SGA group than in the NCUG-SGA group. Correlation analysis showed that the increase in SCFAs was related to the decrease in *Lactobacillus*, *Bilophila*, *Odoribacter*, and *Oscillospira*. As the main metabolite of the intestinal microbiota, SCFAs act as intermediaries between the gut microbiota and the host, and their composition is closely related to the intestinal microbiota. Numerous studies have demonstrated that SCFAs play a positive role in the energy metabolism of mammals. After absorption, SCFAs could be used as energy sources for the host and for regulating energy metabolism (36). In the human body, SCFAs provide about 10% of the daily caloric requirement (37). SCFAs are transported to the liver through the portal vein where butyric acid could stimulate the expression of genes associated with gluconeogenesis (38).Butyric acid has also been shown to be metabolized by acetyl CoA for the synthesis of fatty acids, cholesterol and ketone body through acetyl-CoA metabolism, thereby providing specific substrates for lipid biosynthesis (39). Butyric acid and propionic acid also act as ligands for G protein-coupled receptors Gpr41 and Gpr43 and affect the body's metabolism (40–42). Moreover, changes in SCFA levels may contribute to metabolic disorders such as insulin resistance and obesity. In our study, the increase in SCFA levels may be associated with the metabolic disorders during CUG in SGA. It has to be mentioned that the fecal SCFAs are only a fraction of the total SCFAs and thus changes of fecal SCFAs may not accurately reflect the changes in total SCFAs (37). In addition, it has been proposed that increased SCFAs contributes to the development of obesity, this remains controversial (43).

The metabolism of SCFAs depend on the enterohepatic circulation. By comparing the transcriptome sequences of rat livers in the NCUG-SGA and CUG-SGA groups, we found that the DEGs were mainly involved in insulin response, and carbohydrate, protein, and fatty acid metabolism. Among them, Igf2 was significantly reduced in the CUG group. Studies have found that lower Igf2 concentration is associated with weight gain and obesity (44). Murphy et al. have reported a case of a patient harboring an Igf2 defect combined with insulin resistance and lipid metabolism disorders (45). The Igf2 imprinting gene is very close to the gene encoding insulin on human chromosome 11p, which may play an important role in regulating the body weight and fat in children and adults (26, 46–50). *In vivo* assay showed that decreased Igf2 expression occurred with fat deposition and obesity, which may not be caused by an increase in food intake, but more likely by changes in energy homeostasis (51).

PPI networks suggested that Igf2 may interact with Hgf and Mmp14. Hgf stimulates glucose uptake and metabolism in mouse skeletal muscles and plays an important role in insulin resistance (27). In humans, serum Hgf is significantly higher in patients with obesity, metabolic syndrome, and diabetes compared to healthy subjects (52–54). A prospective study revealed that elevation of serum Hgf is significantly correlated with insulin resistance (27). Mmp14, Igf2, and Hgf are members of MAPK pathway. The circulating glucose and triglyceride levels in Mmp14-knockout mice decreased by 50% compared to levels in wild type mice, as demonstrated by reduced tissue glycogen and lipid levels, and plasma glucose and triglycerides (28). Therefore, we speculated that Igf2, Hgf, and Mmp14 may be involved in the metabolic changes in CUG in SGA. Correlation analysis showed that the expression levels of Igf2, Hgf and Mmp14 were associated with the abundance of *Lactobacillus* and SCFA contents. It is possible that the metabolites of *Lactobacillus* and other SCFAs are detected by the host, leading to altered expressions of Igf2, Hgf and Mmp14, which then regulate the host blood glucose and triglyceride levels and insulin sensitivity in SGA. The detailed mechanism needs further research.

In the present study, we found that *Lactobacillus* may affect the host's body weight and insulin and cholesterol levels by regulating SCFAs during the catch-up process in SGA rats. Changes in *Lactobacillus* levels were associated with increased levels of SCFAs. Following transportation into the liver *via* the enterohepatic circulation, SCFA may be related to the decreasing Igf2, associated with insulin resistance and lipid metabolism disorder. Although the relationships between the microbiota, SCFAs and liver transcriptomes are based on computational analysis and predictions, the consistent findings combining three different profiles of the SGA rats strengthened our study. These findings provide a theoretical basis for elucidating the metabolism and developing novel therapies in SGA patients, especially those with CUG.

## Data Availability Statement

The original contributions presented in the study are publicly available. This data can be found here: NCBI PRJNA679705.

## Ethics Statement

The animal study was reviewed and approved by the Animal Experimental Ethics Committee of Ruijin Hospital, Shanghai Jiao Tong University School of Medicine.

## Author Contributions

ZD and XW proposed the initial experimental ideas. JA, ZD, YX, WL, and XW developed the experimental design. JA, JW, LL, and LC conducted the experiments. JA, ZD, XW, and LG wrote the article. All authors contributed to the article and approved the submitted version.

## Conflict of Interest

The authors declare that the research was conducted in the absence of any commercial or financial relationships that could be construed as a potential conflict of interest.

## References

[1] Lee PA Chernausek SD Hokken-Koelega AC Czernichow P. International Small for Gestational Age Advisory Board consensus development conference statement: management of short children born small for gestational age, April 24-October 1, 2001. Pediatrics (2003) 111:1253–61. 10.1542/peds.111.6.1253 12777538

[2] Rapaport R Tuvemo T. Growth and growth hormone in children born small for gestational age. Acta Paediatr (2005) 94:1348–55. 10.1111/j.1651-2227.2005.tb01801.x 16263627

[3] Hong YH Chung S. Small for gestational age and obesity related comorbidities. Ann Pediatr Endocrinol Metab (2018) 23:4–8. 10.6065/apem.2018.23.1.4 29609443 PMC5894558

[4] Yadav S Rustogi D. Small for gestational age: growth and puberty issues. Indian Pediatr (2015) 52:135–40. 10.1007/s13312-015-0588-z 25691182

[5] Zanelli SA Rogol AD. Short children born small for gestational age outcomes in the era of growth hormone therapy. Growth Horm IGF Res (2018) 38:8–13. 10.1016/j.ghir.2017.12.013 29291885

[6] Mericq V Ong KK Bazaes R Peña V Avila A Salazar T. Longitudinal changes in insulin sensitivity and secretion from birth to age three years in small- and appropriate-for-gestational-age children. Diabetologia (2005) 48:2609–14. 10.1007/s00125-005-0036-z 16283238

[7] Eriksson JG Forsén T Tuomilehto J Winter PD Osmond C Barker DJ. Catch-up growth in childhood and death from coronary heart disease: longitudinal study. Bmj (1999) 318:427–31. 10.1136/bmj.318.7181.427 PMC27731 9974455

[8] Bavdekar A Yajnik CS Fall CH Bapat S Pandit AN Deshpande V. Insulin resistance syndrome in 8-year-old Indian children: small at birth, big at 8 years, or both? Diabetes (1999) 48:2422–9. 10.2337/diabetes.48.12.2422 10580432

[9] Saenger P Reiter E. Genetic factors associated with small for gestational age birth and the use of human growth hormone in treating the disorder. Int J Pediatr Endocrinol (2012) 2012:12. 10.1186/1687-9856-2012-12 22587301 PMC3511163

[10] Woods KA Camacho-Hübner C Savage MO Clark AJ. Intrauterine growth retardation and postnatal growth failure associated with deletion of the insulin-like growth factor I gene. N Engl J Med (1996) 335:1363–7. 10.1056/nejm199610313351805 8857020

[11] Morgan AR Thompson JM Murphy R Black PN Lam WJ Ferguson LR. Obesity and diabetes genes are associated with being born small for gestational age: results from the Auckland Birthweight Collaborative study. BMC Med Genet (2010) 11:125. 10.1186/1471-2350-11-125 20712903 PMC2928774

[12] Sharma D Sharma P Shastri S. Genetic, metabolic and endocrine aspect of intrauterine growth restriction: an update. J Matern Fetal Neonatal Med (2017) 30:2263–75. 10.1080/14767058.2016.1245285 27718783

[13] Backhed F Ding H Wang T Hooper LV Koh GY Nagy A. The gut microbiota as an environmental factor that regulates fat storage. Proc Natl Acad Sci USA (2004) 101:15718–23. 10.1073/pnas.0407076101 PMC524219 15505215

[14] Turnbaugh PJ Ley RE Mahowald MA Magrini V Mardis ER Gordon JI. An obesity-associated gut microbiome with increased capacity for energy harvest. Nature (2006) 444:1027–31. 10.1038/nature05414 17183312

[15] Kootte RS Levin E Salojarvi J Smits LP Hartstra AV Udayappan SD. Improvement of Insulin Sensitivity after Lean Donor Feces in Metabolic Syndrome Is Driven by Baseline Intestinal Microbiota Composition. Cell Metab (2017) 26:611–9 e6. 10.1016/j.cmet.2017.09.008 28978426

[16] McNeil NI. The contribution of the large intestine to energy supplies in man. Am J Clin Nutr (1984) 39:338–42. 10.1093/ajcn/39.2.338 6320630

[17] Rahat-Rozenbloom S Fernandes J Gloor GB Wolever TM. Evidence for greater production of colonic short-chain fatty acids in overweight than lean humans. Int J Obes (Lond) (2014) 38:1525–31. 10.1038/ijo.2014.46 PMC3970979 24642959

[18] Schwiertz A Taras D Schäfer K Beijer S Bos NA Donus C. Microbiota and SCFA in lean and overweight healthy subjects. Obes (Silver Spring) (2010) 18:190–5. 10.1038/oby.2009.167 19498350

[19] Fernandes J Su W Rahat-Rozenbloom S Wolever TM Comelli EM. Adiposity, gut microbiota and faecal short chain fatty acids are linked in adult humans. Nutr Diabetes (2014) 4:e121. 10.1038/nutd.2014.23 24979150 PMC4079931

[20] Teixeira TF Grześkowiak Ł Franceschini SC Bressan J Ferreira CL Peluzio MC. Higher level of faecal SCFA in women correlates with metabolic syndrome risk factors. Br J Nutr (2013) 109:914–9. 10.1017/s0007114512002723 23200109

[21] Wang Y Zhu W Chen L Liang L. Early Growth Hormone Intervention Improves Glucose Metabolism in Adult Rats Born Small for Gestational Age. Exp Clin Endocrinol Diabetes (2020) 128:125–32. 10.1055/a-0723-3544 30257265

[22] Yee JK Lee WN Ross MG Lane RH Han G Vega J. Peroxisome proliferator-activated receptor gamma modulation and lipogenic response in adipocytes of small-for-gestational age offspring. Nutr Metab (Lond) (2012) 9:62. 10.1186/1743-7075-9-62 22726273 PMC3495639

[23] Segata N Izard J Waldron L Gevers D Miropolsky L Garrett WS. Metagenomic biomarker discovery and explanation. Genome Biol (2011) 12:R60. 10.1186/gb-2011-12-6-r60 21702898 PMC3218848

[24] Bolyen E Rideout JR Dillon MR Bokulich NA Abnet CC Al-Ghalith GA. Reproducible, interactive, scalable and extensible microbiome data science using QIIME 2. Nat Biotechnol (2019) 37:852–7. 10.1038/s41587-019-0209-9 PMC7015180 31341288

[25] Zheng X Qiu Y Zhong W Baxter S Su M Li Q. A targeted metabolomic protocol for short-chain fatty acids and branched-chain amino acids. Metabolomics (2013) 9:818–27. 10.1007/s11306-013-0500-6 PMC3756605 23997757

[26] Gu D O’Dell SD Chen XH Miller GJ Day IN. Evidence of multiple causal sites affecting weight in the IGF2-INS-TH region of human chromosome 11. Hum Genet (2002) 110:173–81. 10.1007/s00439-001-0663-5 11935324

[27] Tsukagawa E Adachi H Hirai Y Enomoto M Fukami A Ogata K. Independent association of elevated serum hepatocyte growth factor levels with development of insulin resistance in a 10-year prospective study. Clin Endocrinol (Oxf) (2013) 79:43–8. 10.1111/j.1365-2265.2012.04496.x 22788978

[28] Mori H Bhat R Bruni-Cardoso A Chen EI Jorgens DM Coutinho K. New insight into the role of MMP14 in metabolic balance. PeerJ (2016) 4:e2142. 10.7717/peerj.2142 27478693 PMC4950575

[29] Slover CM Danziger L. Lactobacillus: a Review. Clin Microbiol Newslett (2008) 30:23–7. 10.1016/j.clinmicnews.2008.01.006

[30] Riboulet-Bisson E Sturme MH Jeffery IB O’Donnell MM Neville BA Forde BM. Effect of Lactobacillus salivarius bacteriocin Abp118 on the mouse and pig intestinal microbiota. PloS One (2012) 7:e31113. 10.1371/journal.pone.0031113 22363561 PMC3281923

[31] Hou YP He QQ Ouyang HM Peng HS Wang Q Li J. Human Gut Microbiota Associated with Obesity in Chinese Children and Adolescents. BioMed Res Int (2017) 2017:7585989. 10.1155/2017/7585989 29214176 PMC5682041

[32] Crovesy L Ostrowski M Ferreira D Rosado EL Soares-Mota M. Effect of Lactobacillus on body weight and body fat in overweight subjects: a systematic review of randomized controlled clinical trials. Int J Obes (Lond) (2017) 41:1607–14. 10.1038/ijo.2017.161 28792488

[33] Jiang J Feng N Zhang C Liu F Zhao J Zhang H. Lactobacillus reuteri A9 and Lactobacillus mucosae A13 isolated from Chinese superlongevity people modulate lipid metabolism in a hypercholesterolemia rat model. FEMS Microbiol Lett (2019) 366(24):fnz254. 10.1093/femsle/fnz254 31855230

[34] Ding W Shi C Chen M Zhou J Long R Guo X. Screening for lactic acid bacteria in traditional fermented Tibetan yak milk and evaluating their probiotic and cholesterol-lowering potentials in rats fed a high-cholesterol diet. J Funct Foods (2017) 32:324–32. 10.1016/j.jff.2017.03.021

[35] Drissi F Raoult D Merhej V. Metabolic role of lactobacilli in weight modification in humans and animals. Microb Pathog (2017) 106:182–94. 10.1016/j.micpath.2016.03.006 27033001

[36] DiBaise JK Zhang H Crowell MD Krajmalnik-Brown R Decker GA. Rittmann BE. Gut microbiota and its possible relationship with obesity. Mayo Clin Proc (2008) 83:460–9. 10.4065/83.4.460 18380992

[37] den Besten G van Eunen K Groen AK Venema K Reijngoud DJ Bakker BM. The role of short-chain fatty acids in the interplay between diet, gut microbiota, and host energy metabolism. J Lipid Res (2013) 54:2325–40. 10.1194/jlr.R036012 PMC3735932 23821742

[38] Macfarlane GT Macfarlane S. Fermentation in the human large intestine: its physiologic consequences and the potential contribution of prebiotics. J Clin Gastroenterol (2011) 45 Suppl:S120. 10.1097/MCG.0b013e31822fecfe 21992950

[39] Ji X Zhou F Zhang Y Deng R Xu W Bai M. Butyrate stimulates hepatic gluconeogenesis in mouse primary hepatocytes. Exp Ther Med (2019) 17:1677–87. 10.3892/etm.2018.7136 PMC6364177 30783436

[40] Brown AJ Goldsworthy SM Barnes AA Eilert MM Tcheang L Daniels D. The Orphan G protein-coupled receptors GPR41 and GPR43 are activated by propionate and other short chain carboxylic acids. J Biol Chem (2003) 278:11312–9. 10.1074/jbc.M211609200 12496283

[41] Le Poul E Loison C Struyf S Springael JY Lannoy V Decobecq ME. Functional characterization of human receptors for short chain fatty acids and their role in polymorphonuclear cell activation. J Biol Chem (2003) 278:25481–9. 10.1074/jbc.M301403200 12711604

[42] Xiong Y Miyamoto N Shibata K Valasek MA Motoike T Kedzierski RM. Short-chain fatty acids stimulate leptin production in adipocytes through the G protein-coupled receptor GPR41. Proc Natl Acad Sci USA (2004) 101:1045–50. 10.1073/pnas.2637002100 PMC327148 14722361

[43] Canfora EE Jocken JW Blaak EE. Short-chain fatty acids in control of body weight and insulin sensitivity. Nat Rev Endocrinol (2015) 11:577–91. 10.1038/nrendo.2015.128 26260141

[44] Sandhu MS Gibson JM Heald AH Dunger DB Wareham NJ. Low circulating IGF-II concentrations predict weight gain and obesity in humans. Diabetes (2003) 52:1403–8. 10.2337/diabetes.52.6.1403 12765950

[45] Murphy R Baptista J Holly J Umpleby AM Ellard S Harries LW. Severe intrauterine growth retardation and atypical diabetes associated with a translocation breakpoint disrupting regulation of the insulin-like growth factor 2 gene. J Clin Endocrinol Metab (2008) 93:4373–80. 10.1210/jc.2008-0819 18728168

[46] Le Stunff C Fallin D Bougnères P. Paternal transmission of the very common class I INS VNTR alleles predisposes to childhood obesity. Nat Genet (2001) 29:96–9. 10.1038/ng707 11528401

[47] Dunger DB Ong KK Huxtable SJ Sherriff A Woods KA Ahmed ML. Association of the INS VNTR with size at birth. ALSPAC Study Team. Avon Longitudinal Study of Pregnancy and Childhood. Nat Genet (1998) 19:98–100. 10.1038/ng0598-98 9590300

[48] Lindsay RS Kobes S Knowler WC Hanson RL. Genome-wide linkage analysis assessing parent-of-origin effects in the inheritance of birth weight. Hum Genet (2002) 110:503–9. 10.1007/s00439-002-0718-2 12073022

[49] Roth SM Schrager MA Metter EJ Riechman SE Fleg JL Hurley BF. IGF2 genotype and obesity in men and women across the adult age span. Int J Obes Relat Metab Disord (2002) 26:585–7. 10.1038/sj.ijo.0801927 12075589

[50] Rice T Chagnon YC Pérusse L Borecki IB Ukkola O Rankinen T. A genomewide linkage scan for abdominal subcutaneous and visceral fat in black and white families: The HERITAGE Family Study. Diabetes (2002) 51:848–55. 10.2337/diabetes.51.3.848 11872690

[51] Jones BK Levorse J Tilghman SM. Deletion of a nuclease-sensitive region between the Igf2 and H19 genes leads to Igf2 misregulation and increased adiposity. Hum Mol Genet (2001) 10:807–14. 10.1093/hmg/10.8.807 11285246

[52] Rehman J Considine RV Bovenkerk JE Li J Slavens CA Jones RM. Obesity is associated with increased levels of circulating hepatocyte growth factor. J Am Coll Cardiol (2003) 41:1408–13. 10.1016/s0735-1097(03)00231-6 12706940

[53] Hiratsuka A Adachi H Fujiura Y Yamagishi S Hirai Y Enomoto M. Strong association between serum hepatocyte growth factor and metabolic syndrome. J Clin Endocrinol Metab (2005) 90:2927–31. 10.1210/jc.2004-1588 15713721

[54] Rajpathak SN Wassertheil-Smoller S Crandall J Liu S Ho GY. Hepatocyte growth factor and clinical diabetes in postmenopausal women. Diabetes Care (2010) 33:2013–5. 10.2337/dc10-0710 PMC2928353 20519660

