# Supplementary material for: The Impact of Gut Microbiome on Metabolic Disorders During Catch-Up Growth in Small-for-Gestational-Age
Source: Front Endocrinol (Lausanne). 2021 Mar 4;12:630526. doi: 10.3389/fendo.2021.630526 (PMC7970190; doi:10.3389/fendo.2021.630526)
Supplement: Supplementary file 1 [file Table_1.docx]

Supplementary Material

**Supplementary Table1**

Sequences of primers used for qRT-PCR

| Gene ID | | Sequence |
| --- | --- | --- |
| Igf2 | Forward | ATCAGGCGGGAGCAAGT |
|  | Reverse | TGGGGTGCTGAGGAGTG |
| Hgf | Forward | CTGAGGAGGCTGGAGATG |
|  | Reverse | GATAGGGGCTGGGACTTC |
| Mmp14 | Forward | GGGGCTACACTGGATTGA |
|  | Reverse | TCACCTTGCCTTGATGGT |
| Gapdh | Forward | ATGGCTACAGCAACAGGGT |
|  | Reverse | TTATGGGGTCTGGGATGG |
